# Study on the Genus *Drymaria* (Caryophyllaceae)—A New Species from North-East India

**DOI:** 10.3390/plants13233378

**Published:** 2024-11-30

**Authors:** Sindhu Arya, Harsh Singh, Kalarikkal Walsan Vishnu, Duilio Iamonico

**Affiliations:** 1 PG & Research Department of Botany, PSG College of Arts & Science, Tamil Nadu 641014, India; aryasindu001@gmail.com; 2 Department of Botany, Centre for Advanced Studies in Botany, North-Eastern Hill University, Shillong 793022, India; harshchamlegi@gmail.com; 3 Medicinal Plant Research Section, Regional Ayurveda Research Centre, Dimapur 797112, India; vishnuwalsan@gmail.com; 4 Department of Environmental Biology, University of Rome Sapienza, Piazzale Aldo Moro 5, 00185 Rome, Italy

**Keywords:** *Caryophyllales*, North-East India, typification

## Abstract

A new species of *Drymaria* from the Kohima District (Nagaland of North-East India) is described and illustrated based on both molecular data (the ITS region of nuclear DNA) and morphometric analyses (clustering, PCA, DA, and MANOVA). The new species resembles *D.* cordata but differs with respect to the shape of the sepal (oblong, incurved at the margin vs. lanceolate, not incurved at the margin), petals (oblong or linear vs. ovate–lanceolate), and bract (foliaceous, glabrous, non-prominent vs. non-foliaceous, pubescent, and prominent). For nomenclatural purposes, the typification of *Drymaria villosa* by Duke (in 1961) was corrected according to Art. 9.10 of the Shenzhen Code.

## 1. Introduction

Caryophyllaceae Juss. is one of the most diverse families in the order Caryophyllales, comprising approximately 100 genera and 3000 species, and is mainly distributed in north-temperate, montane, and alpine areas with a center of diversity in the Eastern Mediterranean and Irano-Turanian regions, while its presence in the tropics and the Southern Hemisphere is limited and mostly at higher elevations [1,2,3]. The family is monophyletic, as circumscribed by Bittrich [4], but the traditional classification into three subfamilies [4,5], which is based on stipule, petal, sepal, and fruit features, does not provide monophyletic groups and should be replaced with the tribe-based scheme [1,2]. According to *The Caryophyllales Network* [6], 11 tribes can be recognized at the moment: Alsineae Lam. & DC. (with 15 genera), Arenarieae Kitt. (3 genera), Caryophylleae Lam. & DC. (14 genera), Corrigioleae Dumort. (2 genera), Eremogoneae Rabeler & W.L.Wagner (3 genera), Paronychieae Dumort. (5 genera), Polycarpaeae DC. (25 genera), Sagineae J.Presl (14 genera), Sclerantheae DC. (10 genera, including the recently described genus *Maguirellaria* Iamonico [7]), Sileneae DC. (9 genera), and Sperguleae Dumort. (3 genera); a further 4 genera (*Calycotropis* Turcz., *Dadjoua* Parsa, *Dolophragma* Fenzl, and *Pentastemonodiscus* Rech.f.) are unplaced.

According to phylogenetic analyses of the whole family [1,2,7], the genus *Drymaria* Willd. ex Schultes belongs to the tribe Polycarpeae, a well-supported clade which is basal to a large group including many other tribes of Caryophyllaceae, i.e., Sperguleae, Sclerantheae/Sagineae, and Ermogoneae/Caryophylleae/Sileneae. This genus is represented by 57 species native mainly to tropical and subtropical regions of the world [8].

*Drymaria* is a poorly known genus. In fact, the only comprehensive taxonomic revision was published about 70 years ago by Duke [9], who recognized 48 species and proposed a classification of the genus by recognizing 17 series. Hartman [10], Villarreal and Estrada [11], and Montesinos-Tubeé et al. [12] stated that Duke’s series were not validly published, being *nomina nuda*. However, by checking Duke’s paper, it transpires that each series is in fact valid from a nomenclatural point of view (see Art. 38.1 of the *Shenzhen Code* [13]), having an own description or, for monospecific series, a description of the single species [9] (pagg. 186, 189, 193, 200, 201, 204, 210, 212, 216, 220, 222, 223, 230, 240, 245). No molecular study on all the species belonging to *Drymaria* has been published until now.

The flora of India includes three *Drymaria* species, i.e., *D. cordata* (L.) Willd. ex Schult. [alien in Haryana State (N-India [14]), Assam (NE India [15,16]), Upper Siang District in Arunachal Pradesh (NE India [17])], *D. diandra* Blume (hillock regions of India, from the Western to the Eastern Himalaya [18]), and *D. villosa* Schltdl. & Cham. (Eastern, Western, and Central Himalaya and Southern India [18,19,20]), all occurring in the Kerala region.

As a part of ongoing studies of the family Caryophyllaceae in India (see, e.g., [21,22]), we found an interesting population of *Drymaria* from Kohima District (Nagaland, NE India). Based on both morphometric and molecular analyses, as well as a critical examination of the literature, we reached the conclusion that the population from Nagaland can be described as a species new to science.

## 2. Material and Methods

### 2.1. Morphometric Data

The work was carried out via field surveys in Nagaland during the monsoon season (August to November) of the years 2022–2023, examination of herbarium specimens housed at BM, CALI, CAL, P, UCBD, and TBGT (acronyms according to [23]), and analysis of the relevant literature (cited below throughout the text).

The data matrix [characters (columns) × individuals (=specimens; rows)] was processed by means of the software NCSS 2007 ver. 01.1.21 (Kaysville, UT, USA). Seven populations, 14–19 individuals per population (we were not able to collect more individuals per population since they were too reduced) were investigated (Table 1) and 21 characters (1 discrete, 20 continuous; Table 2) were measured in 90 individuals (30 for each native species, i.e., *Drymaria diandra*, *D. villosa*, and the new population, hereafter named “*Drymaria* sp.”). After a preliminary recognition of the morphological characters of *Drymaria* throughout its distribution area (herbarium specimens), we decided to collect populations in Nagaland State, where more similarities between them were expected. An explorative cluster analysis was performed by the UPGMA method. The data matrix was also submitted to ordination (Principal Component Analysis, PCA; a correlation matrix of only continuous characters) and Discriminant Analysis (DA). The DA was performed using the first three components derived from the PCA, which explain about 85% of the total variability. As a supervised technique, we performed the DA on groups classified as species; the matrix of actual/predicted groups was analyzed by comparing the values among these groups, especially regarding the diagonal, whose values reveal the matching of actual and predicted observations for each group. The value of correct classification reported in the results is the classification accuracy achieved by the actual discriminant functions over what is expected. A multivariate analysis of variance (MANOVA) was also performed to test the significance of differences between response (=dependent) variables (morphological characters) and factor variables (=taxonomic groups).

Photographs of specimens were taken using a Canon 1500 camera and, for macromorphology, they were taken using a Carl Zeiss camera. Seed and pollen grains were examined using SEM (Carl Zeiss, Oberkochen, Germany).

### 2.2. Molecular Analysis

Fresh leaf samples weighing 50 mg were crushed in liquid nitrogen using a sterile mortar and pestle, yielding a fine powder. DNA extraction was carried out using a Plant/Fungi DNA isolation kit or the CTAB method [24]. PCR reactions were carried out using a Thermo Fisher thermal cycler (Applied BioSystems, Waltham, MA, USA) under the following conditions: initial denaturation at 94 °C for 5 min, followed by 40 cycles of denaturation at 94 °C for 15 s, annealing at 60 °C for 20 s and extension at 72 °C for 1:00 min, with a final extension at 72 °C for 5 min. The PCR reactions were performed in a 20 μL volume beaker containing Emarald PCR Master mix at 1× final concentration [25], 500 ng of genomic DNA, and 0.2 micromoles of primers. The ITS region was used for phylogenetic analysis of the new species.

The obtained sequences were compared with other available sequences in the NCBI database using the Basic Local Alignment Search Tool (BLAST) [26]. DNA sequences of the rbcL region were submitted to NCBI/GENBANK (Table 3). Molecular phylogenetic analysis for the rbcL gene was carried out by using the MEGA X software var. 11.0.10. The phylogenetic tree was constructed using the neighbor joining (NJ) method with a Jukes–Cantor substitution model [27], selecting bootstrap values with the resampling number set to 1000 [28] to view and download the constructed phylogenetic tree. All positions containing gaps and missing data were excluded during phylogenetic analysis.

**Figure 1:**
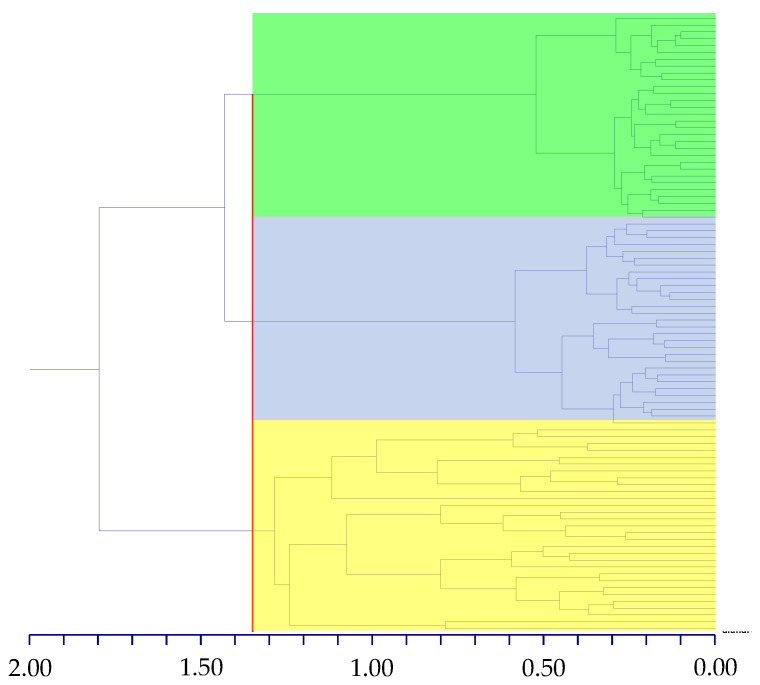
Dendrogram (UPGMA method) showing the relationships between the specimens examined in the present study (the vertical axis is dissimilarity). The red line refers to the cutoff dissimilarity point (1.35). Clades: *Drymaria* sp. (green), *D. villosa* (blue), and *D. diandra* (yellow).

We considered the molecular studies by Harbaugh et al., Greenberg & Donoghue, and Dillenberger & Kadereit [1,2,7] to select genera related to *Drymaria*, i.e., the following members of the tribe Polycarpeae: *Achyronychia cooperi* A.Gray, *Cardionema ramosissimum* (Weinm.) A.Nelson & J.F.Macbr., *Dicheranthus plocamoides* Webb, *Illecebrum verticillatum* L., Loeflingia hispanica L., *Sphaerocoma aucheri* Boiss., *Spergula arvensis* L., *Spergularia media* (L.) C.Presl, *Spergularia rubra* (L.) J.Presl & C.Presl, *Polycarpon tetraphyllum* L. subsp. *tetraphyllum*, *Pteranthus dichotomus* Forssk., *Pycnophyllum bryoides* (Phil.) Rohrb., *Pycnophyllum spathulatum* Mattf., and *Scopulophila rixfordii* (Brandegee) Munz & I.M.Johnst. Further, other groups were selected based on Figure 1B–D by Greenberg and Donoghue [2], i.e., the generitypes of *Dianthus* (*Dianthus caryophyllus* L. from the tribe Caryophylleae), *Stellaria* L. (*Stellaria holostea* L. for Alsineae Lam. & DC.), *Arenaria* L. (*Arenaria serpyllifolia* L. for Arenarieae Kitt.), *Sleranthus* L. (*Sleranthus annuus* L. for Slerantheae), *Sagina* L. (*Sagina procumbens* L. for Sagineae), and *Silene* L. (*Silene gallica* L. for Sileneae).

## 3. Results and Discussion

### 3.1. Morphometric Data

The analyses of the characters allow the recognition of three distinct groups.

Concerning the hierarchical clustering (Figure 1), the cutoff dissimilarity point (1.35) defines three clusters, with each one comprising specimens referring to single species, i.e., *Drymaria diandra*, *D. villosa*, and *Drymaria* sp.

**Figure 2:**
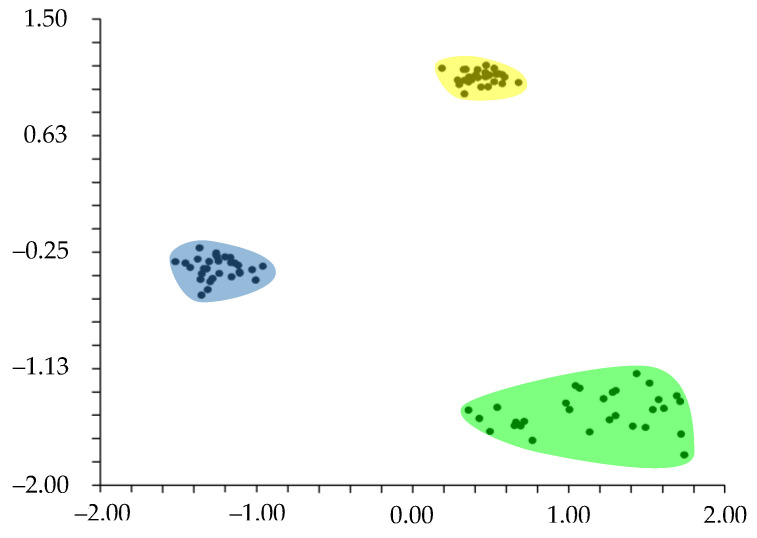
Ordination (PCA) based on quantitative morphological characters measured in the present study (component1 vs. component2). Groups: *Drymaria* sp. (yellow), *D. diandra* (blue), and *D. villosa* (green).

The PCA analysis shows that the cumulative percentage of eigenvalues for the first two axes is 78.76%, with the contribution given by the first component being 58.06% and that by the second component being 20.70%. The component1 vs. component2 graphs show three well-separated groups along the first component, whereas, along component2, two groups are partially overlapped (Figure 2). These three groups correspond to the populations of *Drymaria diandra*, *D. villosa*, and *Drymaria* sp., as displayed by the clustering. The highest contribution to the first component is given by the following characters: length and width of fruits, length and width of sepals, and length of stamens. The second component is mainly related to the length and width of gynoecium, length of petiole, seed diameter, width of petals, and length of bracts.

**Figure 3:**
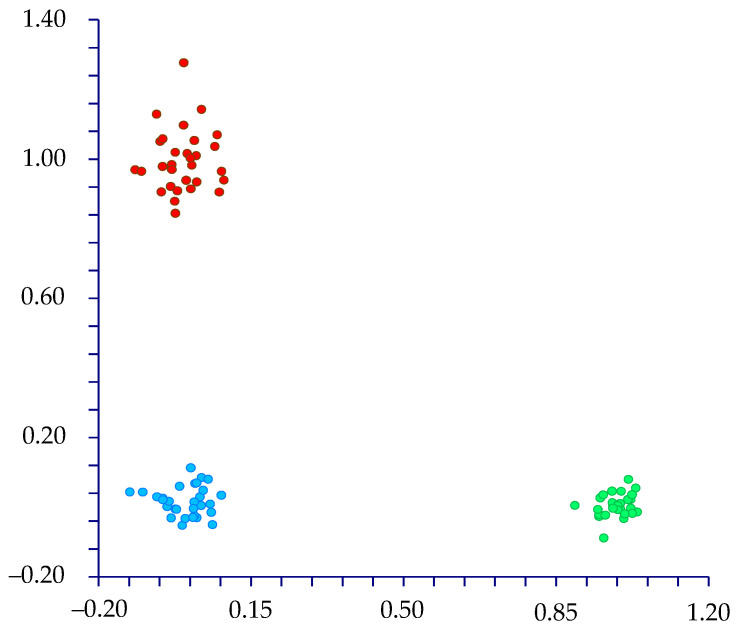
DA. The first two discriminant functions explain 100.0% of the total variation [eigenvalues: 79.7% (1st function) and 20.3% (2nd function)]. Dots: *Drymaria diandra* (red), *D. villosa* (blue), and *Drymaria* sp. (green).

The DA, carried out using the names of the species (three groups: *Drymaria diandra*, *D. villosa*, and *Drymaria* sp.), predicted three clearly separated groups (Figure 3) based on the first two discriminant functions, which explain 100.0% of the total variation [eigenvalues: 79.7% (first function) and 20.3% (second function)]. These groups correspond to *D. diandra*, *D. villosa*, and *Drymaria* sp.

The results of the MANOVA show significant differences at both species and population levels. The probability level is less than 0.000001 for all the statistical tests considered (Wilks’ lambda, Hotelling–Lawley trace, Pillai’s trace, and Roy’s largest root). F-ratios are high, ranging from F = 27.03 to 2020.7 (populations as groups; Table 4) and F = 1639.56 to 4011.09 (species as groups; Table 5).

### 3.2. Molecular Analysis

**Figure 4:**
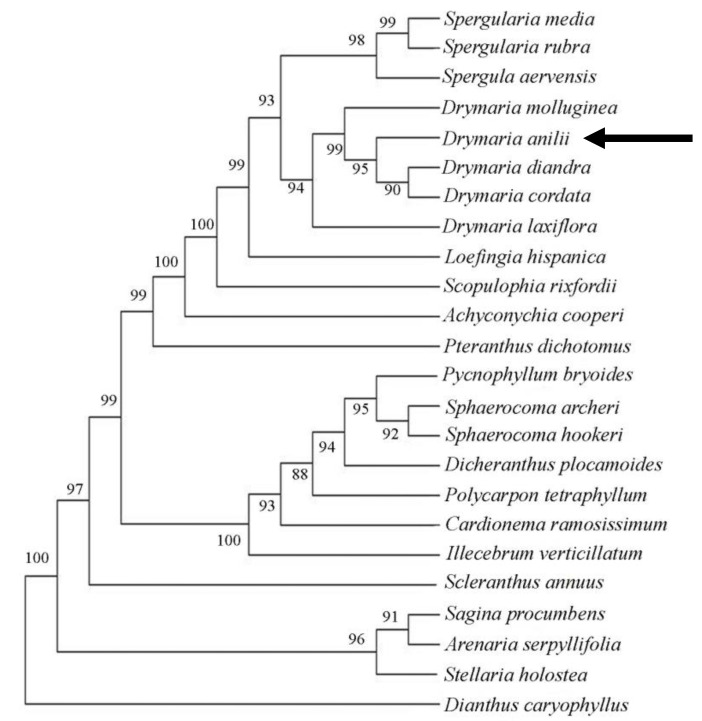
Neighbor joining dendrogram. *Drymaria* sp. is “*Dryamria anilii*” (proposed binomial for the new species; see paragraph “3.3. Taxonomic treatment”) in the dendrogram.

The molecular sequencing ITS region of the nuclear DNA supported *Drymaria* sp. as distinct from the other allied species of *Drymaria* (Figure 4). The new species is separated in the second clade, which comprises *D. cordata*, *D. mollugineae*, *D. grandulosa*, and *D. laxiflora*.

A total of 658 aligned bp of the ITS sequence were considered for comparing *Drymaria* sp. with *D. cordata*. From this, 15 sites distinguished *Drymaria* sp. from *D. cordata*. The out groups are also well separated in the dendrogram. Thus, the results of phylogenetic analysis also support the status of *Stellaria mcclintockiae* as a distinct species.

### 3.3. Taxonomic Treatment

Both the morphological and molecular results obtained in the present research demonstrated that the *Drymaria* populations growing in Kohima District (Nagaland region, NE India) are different to the other Indian *Drymaria* species. Consequently, here, we propose describing the population of *Drymaria* as a new species for science. A complete taxonomic treatment of *Drymaria* taxa occurring in India follows.

**Figure 5:**
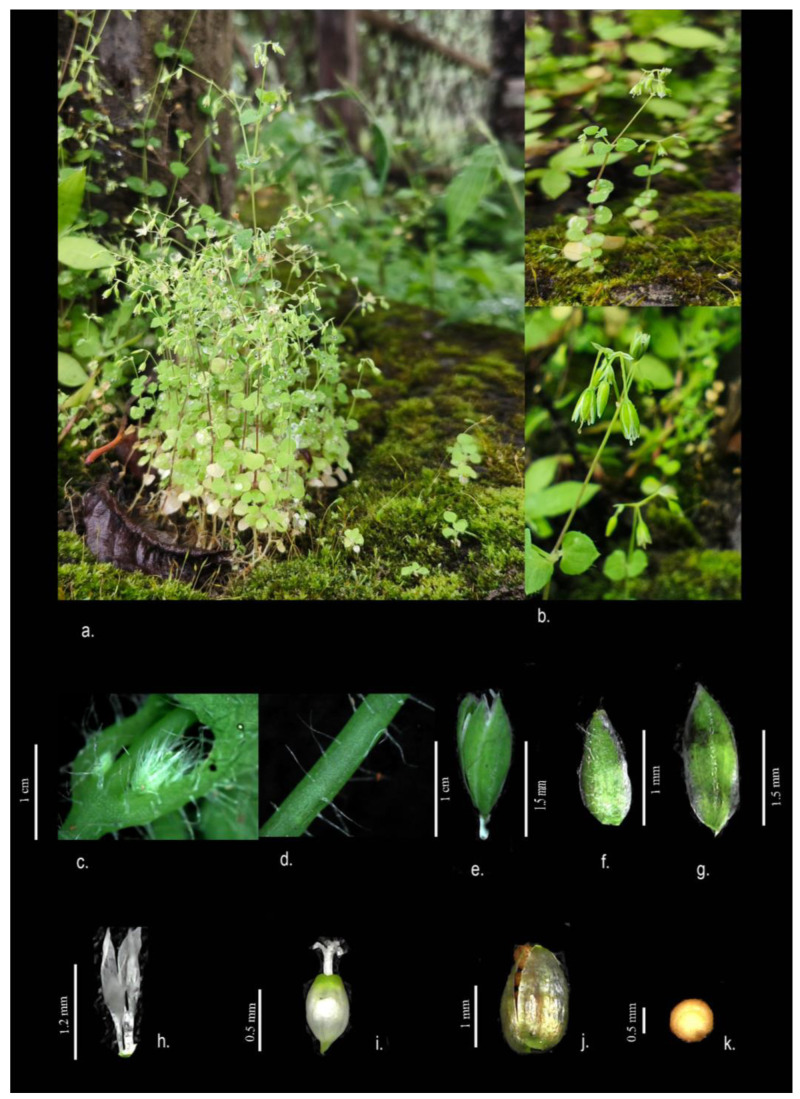
*Drymaria anilii* (**a**) habit; (**b**) detail of inflorescence; (**c**) hairiness at nodes; (**d**) stem; (**e**) closed flower; (**f**) bracteole; (**g**) tepal; (**h**) petal; (**i**) gynoecium; (**j**) fruit; (**k**) seed (photos by Arya Sindhu).

**1.**  ***Drymaria anilii*** S. Arya, Harsh & Iamonico, sp. *nov.* (Figure 5).
  Holotype: INDIA. Nagaland: Kohima District, Kisama Village, 1800 m a.s.l., 25°37′35″ N 94°60′48.9″ E, 20 October 2021, *Arya 730* (DMP!; isotypes: DMP!, RO!).
  **Diagnosis.** *Drymaria anilii* resembles *D. diandra*, from which it differs in terms of inflorescence (sessile in a single terminal flower vs. cymose with 10–15 flowers), bracts (foliaceous, glabrous, 2 cm long vs. non-foliaceous, pubescent, less than 5 mm long) pedicel (glabrous vs. glandular), sepal (obtuse or rounded at the apex, incurved at the margin vs. acute at the apex, not incurved at the margin), petal (obtuse or rounded at the apex and linear vs. acute at the apex and ovate), fruit (ellipsoidal, 6 mm long vs. ovate, 3 mm), pollen (spherical with depressed non-prominent pores vs. ellipsoid with prominent pores), and seed surface architecture (small mamillated spots which form a star-like radiating pattern vs. large spinulate spots that cross each other).

**Description (macromorphology).** An annual herb (therophyte) which is slender, branching from the base, and 10–12 cm long. The root is thin, producing many short horizontal spreading branches, sometimes originating from the lower stem nodes. The stem is prostrate or ascending and erect pubescent with glandular multiseriate trichomes. The leaves are opposite, orbicular to cordate (1.3–2.1 cm × 1.2–1.5 cm), glabrous in the adaxial side, and sparsely pubescent on the abaxial surface; the apex is rounded or acute, and the base is cordate; the petioles are 1–5 mm long, hairy; and the stipules are minute, at 0.1 mm long. There are inflorescences in the reduced cyme (up to three-flowered); the peduncles are 1.5–2.5 mm long and hairy; the bracts are linear to lanceolate and 2 mm long; the pedicels of the flowers are 1–2 mm long and sparsely pubescent; the five sepals are, 1.2–1.4 × 0.6–0.7 mm long, lanceolate, and the apex is acute or obtuse and hairy; the five petals are 1.2–1.3 mm long and bifid (rounded lobes); the stamens are five fewer in number than the sepals with a 0.5 mm long filament and oblong, yellow-brown anthers; the staminode is absent; the ovary is ovoid or globose and 0.7–0.8 mm, and the style is 0.4–0.6 mm long and parted into four at the apex with glands. The capsules are ovoid, 1.7–1.9 mm long, equaling or slightly exceeding the sepals, and opening by four valves to the base. The seeds are brown and reniform.

**Figure 6:**
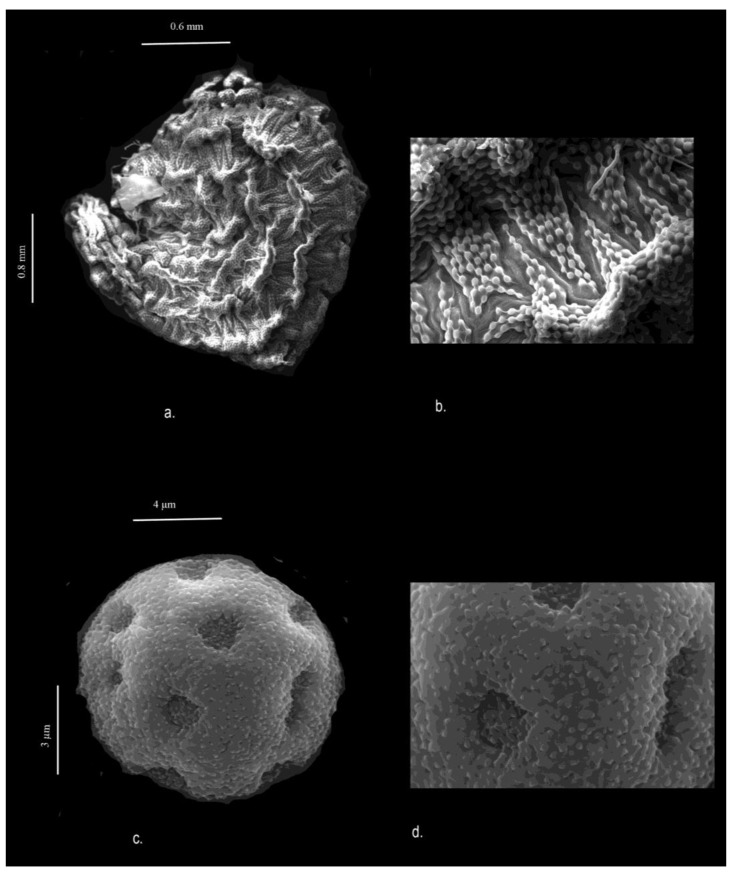
SEM images of *Drymaria anilii*: (**a**) seed; (**b**) magnification of seed surface; (**c**) pollen grain; (**d**) magnification of pollen grain.

**Description (micromorphology; Figure 6).** The pollen grains are spheroidal, poly-pantoporate, and 8.16–10.64 µm in diameter, with 5–6 visible pores. The pore margin or aperture is well demarcated, at about 2 µm. The pores are 3.2–3.3 µm in size. The surface is micro-echinate; the echinates are blunt or appear like dots which are fused. Ektexinous bodies are present inside the pore, minute, and number 4–8 fused, but the apex is sharp and free.

The seeds are reniform with a circular base (0.548–0.765 × 0.614–0.761 mm). The margin is wavy, and the tubercles are short and prominent; small mamillated spots which form a star-like radiating pattern are visible on the surface. The dots form a “V” shape that do not cross each other.

**Etymology.** The species is named in honor of Dr. V.S. Anil Kumar, Principal of Government College Kasargode, Kerala, India, in recognition of his outstanding contributions to the field of plant taxonomy of the Western Ghats. Dr. Anil Kumar is acknowledged as a great teacher and motivator in the field.

**Figure 7:**
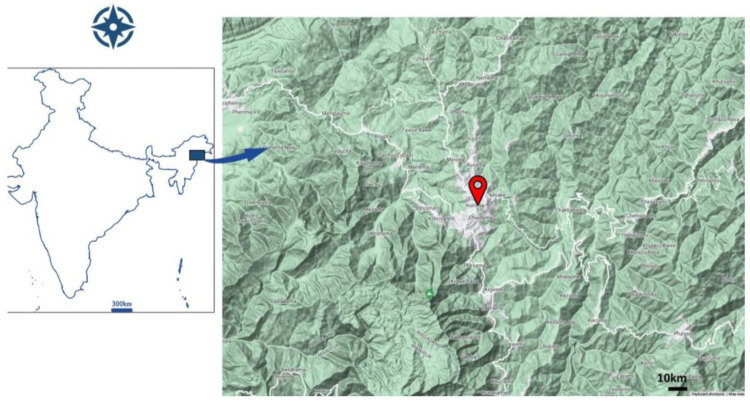
Location of *Drymaria anilii* in NE India.

**Habitat distribution (Figure 7) and phenology.** *Drymaria anilii* is known from only the *locus classicus* (Kohima, Nagaland, NE India at an elevation of 1800 m a.s.l.), where it grows along the muddy slope. Each population spreads over an area of less than 5 km. Flowering and fruiting times are June to November.

**Conservation status.** Even though the plant propagates through vegetative propagation (rooting at the nodes), the number of individuals is very small and they are vulnerable to the severe grazing. There are possibilities of the species occurring in other parts. More extensive studies on the species are required to reach a conclusion on its conservation status. Therefore, by following the IUCN Red List criteria [29], *Drymaria anilii* is assessed as Data Deficient (DD).

**Taxonomic notes.** The three *Drymaria* species occurring in India (*D. cordata*, *D. diandra*, and *D. villosa*) usually have a restricted distribution area and can be found across the Himalayan belts and the hilltops of the Western and Eastern Ghats. Based on the morphology of *D. anilii*, especially its stipulate leaves, terminal solitary cyme, bifid petals, 2–5 stamens, usual three styles, and one or many seeds, it would belong to the series Cordatae [9] (p. 245). The series is represented by five species, four subspecies, and two varieties in Duke’s monograph (*Drymaria gracilis* Schltdl. & Cham., *D. gracilis* subsp. *carinata* (Brandegee) A.J.Duke, *D. glandulosa* C.Presl var. *glandulosa*, *D. glanduosa* var. *galeottiana* (Briq.) A.J.Duke, *D. xerophylla* A.Gray, *D. ladewih* Rusby, *D. cordata* (L.) Willd. ex Roem. & Schult. and *D. diandra* Blume). All these mentioned species, except for *D. cordata* and *D. diandra*, have a distribution in the Americas (the USA, Argentina, and Mexico [8]). Note, however, that a comprehensive molecular study to demonstrate that Duke’s classification is natural is lacking. Consequently, the inclusion of *D. anilli* in the Ser. Cordatae is preliminary and deserves further investigations.

**2.**  ***Drymaria cordata*** (L.) Willd. ex Schult., in J.J.Roemer & J.A.Schultes, Syst. Veg., ed. 15[bis]. 5: 406 (1819) ≡ *Holosteum cordatum* L., Sp. Pl. 1: 88 (1753) ≡ *Cerastium cordatum* (L.) Crantz in Inst. Rei Herb. 2: 400 (1766).
  Lectotype [designated by Burger in Cafferty and Jarvis [30] (p. 1052): [Icon] *Alsine americana nummulariae folio* [31] (t. 11)]; image available at https://bibdigital.rjb.csic.es/viewer/13586/?offset=#page=35&viewer=picture&o=bookmark&n=0&q= (accessed on 26 November 2024).

**3.**  ***Drymaria diandra*** Blume, Bijdr. Fl. Ned. Ind.: 62 (1825) ≡ *Drymaria cordata* subsp. *diandra* (Blume) J.A.Duke in Ann. Missouri Bot. Gard. 48: 253 (1961).
  Lectotype [designated by Mizushima [32] (p. 81)]: INDONESIA. Java, in paludosis montanis, October, *Blume 1549* (L99-143-199, *non vidi fide* Mizushima 1957: 81); isotypes: BM000946422! (image available at https://data.nhm.ac.uk/object/0db637d6-3591-4232-b01a-f014f2715810/1704153600000, accessed on 26 November 2024), MO216778! (image available at https://plants.jstor.org/stable/viewer/10.5555/al.ap.specimen.mo-216778, accessed on 26 November 2024).

**Note.** The isotypes listed above (BM000946422 and MO216778) were not reported by neither Mizushima [32] (p. 81) nor Duke [5] (p. 253).

**4.**  ***Drymaria villosa*** Cham. & Schltdl., Linnaea 5(2): 232–233 (1830) subsp. *villosa* ≡ *Drymaria cordata* var. *villosa* (Cham. & Schltdl.) Rohrb. in C.F.P.von Martius & auct. suc. (eds.), Fl. Bras. 14(2): 260 (1872).
  Lectotype [designated by Duke [9] (p. 226)] as “isotype”, corrected here according to Art. 9.10 of the *Shenzhen Code* [13]): MEXICO. In aquosis prope *Jalapam*, *Chamisso 505* (LECB0000548!, image available at https://plants.jstor.org/stable/viewer/10.5555/al.ap.specimen.lecb0000548, accessed on 24 November 2024).

**Note.** Duke [9] (p. 226) stated “Drymaria villosa Cham. & Schlecht. in Linnaea 5:232. 1830. (HOLOTYPE: Schiede & Deppe 505; in aquosis prope Jalapam, B, probably destroyed; isotype at LE!”. Collections at B were mostly destroyed during World War II, as highlighted by many authors (e.g., [33,34,35,36,37]). According to Art. 9.10 of the Shenzhen Code [13], the term “isotype” used by Duke [9] (p. 226) is to be corrected to lectotype.

### 3.4. A Diagnostic Key of the Members of Drymaria Series Cordatae (Sensu Duke 1961)

1a. Petals equal the sepals; seeds numerous, less than 1 mm, broad………………………………………..2

1b. Petals shorter than sepals; seeds one to many, 0.5–2.0 mm broad………………………………………4

2a. Leaves ovate–reinform; stipules longer than the petiole; petals divided by about half

the length; lobes 2–4, nerved………………………………………………………*D. glandulosa* var. *galeottiva*

2b. Leaves deltoid; long petiole; stipules shorter than the petiole; petals divided by more

than half its length; 1 lobe, nerved………………………………………………………………………………3

3a. Stipules lacerate; seeds coarsely tuberculate; tubercles prominent………….*D. gracilis* subsp. *gracilis*

3b. Stipules entire; seed merely verucate with remote protuberance……………………….subsp. *carinata*

4a. Sepals slightly longer than petals; seeds 0.5–0.8 mm; stipules lacerate…………………………………5

4b. Sepals twice the length of petals; seeds 0.7–2.0 mm; stipules entire…………………………………….6

5a. Flowers in terminal cymes; leaves petiolate; petals bifid;

stamens 3–5…………………………………………………………………………*D. glandulosa* var *glandulosa*

5b. Flowers clustered in the axil of the leaves; petals bifid or ligulate;

stamens 2–3……………………………………………………………………………………………*D. xerophylla*

6a. Leaves deltoid–ovate; stipules entire; seeds 3–5, styles united for half the length…………..*D. ladewii*

6b. Leaves reniform, stipules lacerate, seeds 1–12, style free…………………………………………………7

7a. Flowers campanulate; pedicels glandular; seeds 1–12, broad……………………………………………8

7b. Flowers pyriform; pedicles eglandular; seeds 1–2, broad……………………………………..*D. diandra*

8a. Seeds have small mamillated spots with a star-like radiating pattern; pollen spherical, 

depressed with non-prominent pores……………………………………………………………………*D.anilii*

8b. Seeds have large spinulate spots that cross each other; pollen oblong with prominent 

pores………………………………………………………………………………………………………*D. cordata*

### 3.5. Conclusions

*Drymaria*, a genus belonging to the large tribe Polycarpaeae of the Caryophyllacecae family, is poorly known from a taxonomical point of view. In fact, a recent comprehensive study is lacking, and the last one was published about 70 years ago by Duke [9]. Also, molecular data are few, and those available are included in a wider paper on the whole family [2]. Among the important gaps in morphological and molecular studies of *Drymaria*, there are, e.g., the Andean species [12], but also Asian species (three—*D. cordata*, *D. diandra*, and *D.villosa*—according to the *Plants of the World Online* database [8]) which were not investigated in detail. So, our study, which deals with these three species, represents the first one for the continent. Furthermore, the combined use of both morphometric and molecular techniques appears to have never been considered before us for *Drymaria* and proved to be very useful for analyzing taxonomically critical plant taxa, as highlighted by other authors regarding *Caryophyllales* groups, e.g., *Allmania* R.Br. ex Wight [38], *Dianthus* L. [39], *Limonium* Mill. [40], or *Salicornia* L. [41].

## Figures and Tables

**Table 1:** *Drymaria* populations studied in the present research.

Locality	Taxon
India, Nelliyampathy, Palakkad, Kerala, on muddy slopes	*D. diandra*
India, Pothundi, Palakkad, Kerala, on plain land	*D. diandra*
India, Cheruthoni, Idukki, Kerala, on slopes	*D. diandra*
India, Kohima, Nagaland, on muddy slopes	*Drymaria* sp.
India, Kohima, Nagaland, on slopes	*Drymaria* sp.
India, Adimali, Idukki, Kerala, on muddy slopes	*D. villosa*
India, Minnampara, Palakkad, Kerala, on muddy slopes	*D. villosa*

**Table 2:** Characters measured for the morphometric analysis. The characters labeled with an asterisk are qualitative, and the other ones are quantitative.

1. Height of plant (cm)
2. Length of leaf blade (mm)
3. Width of leaf blade (mm)
4. Length of petiole (mm)
5. Diameter of petiole (mm)
6. Number of flowers per inflorescence *
7. Length of bracteoles (mm)
8. Width of bracteoles (mm)
9. Length of bracts (mm)
10. Width of bracts (mm)
11. Length of sepals (mm)
12. Width of sepals (mm)
13. Length of petals (mm)
14. Width of petals (mm)
15. Length of stamens (mm)
16. Length of gynoecium (mm)
17. Width of gynoecium (mm)
18. Length of fruit (mm)
19. Width of fruit (mm)
20. Minor diameter of seed (mm)
21. Major diameter of seed (mm)

**Table 3:** GenBank accession numbers and taxa selected for molecular analysis.

Sl No.	Species	GenBank Accession Number
1	*Spergularia media*	EU812820
2	*Spergularia rubra*	OQ150721
3	*Spergularia marina*	MG234808
4	*Drymaria diandra*	PP0712821
5	*Loeflingia hispanica*	KX282251
6	*Scopulophia rixfordii*	MF963915
7	*Drymaria anilii*	PP068051
8	*Drymaria cordata*	KF737457
9	*Drymaria molluginea*	JN589128
10	*Drymaria laxiflora*	AY286528
11	*Achyronychia cooperi*	MF964174
12	*Pteranthus dichotomus*	AY936250
13	*Sphaerocoma aucheri*	AJ310979
14	*Sphaerocoma hookeri*	HE586023
15	*Pycnophyllum bryoides*	JN589108
16	*Dicheranthus plocamoides*	KF737513
17	*Polycarpon tetraphyllum*	OQ412391
18	*Cardionema ramosissimum*	MG235912
19	*Illecebrum verticillatum*	KX167481
20	*Scleranthus annuus*	OQ150654
21	*Sagina procumbens*	OR593647
22	*Arenaria serpyllifolia*	MG101851
23	*Stellaria holostea*	KX183996
24	*Dianthus caryophyllus*	MT923226

**Table 4:** MANOVA applied to populations as groups.

Statistic Test	Test Value	F-Ratio	*p* (0.05)
Wilks’ Lambda	0.000176	258.1	0.00000001
Hotelling–Lawley Trace	182.958906	809.76	0.00000001
Pillai’s Trace	1.984377	27.03	0.00000001
Roy’s Largest Root	146.074566	2020.7	0.00000001

**Table 5:** MANOVA applied to species as groups.

Statistic Test	Test Value	F-Ratio	*p* (0.05)
Wilks’ Lambda	0.000194	2008.38	0.00000001
Hotelling–Lawley Trace	175.589622	2458.25	0.00000001
Pillai’s Trace	1.965632	1639.56	0.00000001
Roy’s Largest Root	139.9216	4011.09	0.00000001

## Data Availability

Data are contained within the article.
